# A Case of Purple Urine Bag Syndrome in a Spastic Partial Quadriplegic Male

**DOI:** 10.7759/cureus.552

**Published:** 2016-04-01

**Authors:** Sibghat Tul Llah, Salman Khan, Atman Dave, Amelia Jane A Morrison, Swapna Jain, David Hermanns

**Affiliations:** 1 Department of Medicine, School of Medicine, University of Missouri Kansas City; 2 Medical Education, Saint Luke’s Hospital of Kansas City

**Keywords:** purple urine, purple urine bag syndrome

## Abstract

Purple bag urine syndrome (PUBS) is a benign and unique phenomenon of the urine turning a deep violet color within the urinary catheter tubing and bag. This phenomenon is commonly encountered in patients indicated with long-term catheter placement or, in certain conditions like chronic constipation, alkaline urine, limited ambulation, and, in terms of gender distribution, the female sex, predominates. PUBS gets its name from a unique phenomenon that takes places inside the gut where tryptophan (an amino acid) is metabolized, producing blue and red hues which together emanate a deep violet color. Here, the case of a middle-aged male patient with a suprapubic catheter in situ, following trauma causing spastic partial quadriplegia, is being presented with PUBS due to UTI secondary to Proteus vulgaris. The risk factors, in this case, include chronic constipation and recurrent urinary tract infections (UTIs).​

## Introduction

Purple urine bag syndrome (PUBS) is a rare clinical entity and was first reported in 1978. It is characterized by a purplish discoloration of the urine in patients with urinary tract infections due to specific organisms and generally affects chronically debilitated patients requiring long-term indwelling urinary catheters. The mechanism entails recurrent urinary tract infections with bacteria containing sulphatase and phosphatase enzymes, which result in the formation of pigments indirubin (red) and indigo (blue). These pigments consequently turn the urine purple.​

## Case presentation

A 52-year-old African-American male presented to the emergency department with a one-week history of dark urine and abdominal pain. His past medical history was significant for spastic partial quadriplegia (C5-C7, incomplete) secondary to trauma six years prior, neurogenic bladder with suprapubic catheter placement five years prior, recurrent urinary tract infections (UTI), depression, and chronic constipation. He complained of pain and could localize it in the right lower quadrant of the abdomen with no radiation to any other part of the abdomen. He described the pain as constant, burning in nature, and 8 out of 10 in severity. The pain was associated with fever, chills, and nausea. On query, he denied hematuria, urostomy site discharge, flank pain, vomiting, diarrhea, or bloody stools. The patient had been afflicted by multiple UTIs in the last six months, the causal organisms for which included the Morganella species, Vancomycin-resistant enterococci, and Escherichia coli. His symptoms during this admission were consistent with those of his previous infections. Surgical history included two herniated cervical disc repairs and suprapubic catheter placement. He was not allergic to any drug in particular. His domiciliary medications included baclofen, gabapentin, docusate sodium, glycerin suppository, mirtazapine, and sertraline. He did not report any significant medical diseases running in his family. He did not report any alcohol, tobacco, or illicit drug use. His ambulation was restricted, necessitating bed confinement and the use of a wheelchair due to paraplegia and spastic deforming contractures. On physical exam, his blood pressure was 124/74, he had a pulse rate of 77, and his oral body temperature was 98.4 degrees Fahrenheit. His body mass index was 17.63 kg/m. The remainder of his physical examination was significant for lower extremity contractures. There was no abdominal tenderness and rigidity.

Initial lab work revealed a hemoglobin of 12.4 g/dl, a white blood count of 11.39 Th/uL, and a platelet count of 286 Th/uL. A basic metabolic panel (BMP) showed a sodium at 143 meq/L, potassium at 4.4 meq/L, chloride at 108 meq/L, bicarbonate at 27 meq/L, BUN at 17 mg/dl, and creatinine at 0.7 mg/dl. Blood sugars were at 97 mg/dl. Urinalysis with microscopy revealed purple urine, leukocytes at 21 40/HPF, RBCs at 15 cells/HPF, leukocyte esterase positive, and nitrates positive. Urine was alkalotic with a pH of 8.5 and a specific gravity of 1.010. Empirically intravenous ceftriaxone was initiated on a daily basis.

**Figure 1:**
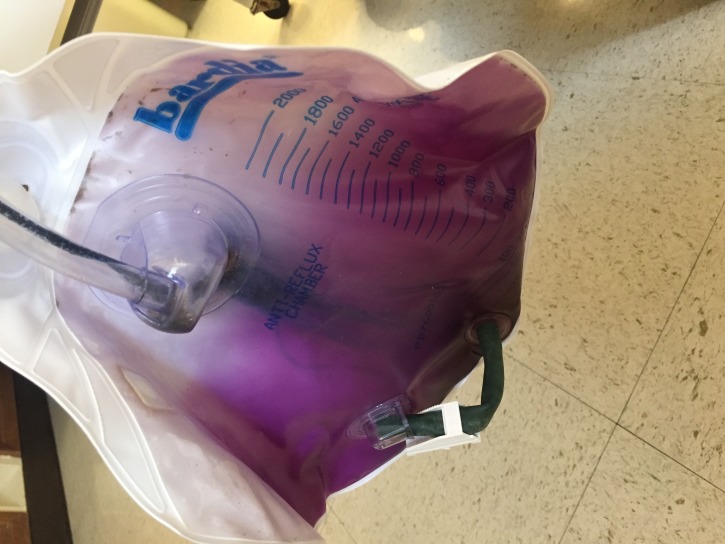
Purple urine in urine bag.

**Figure 2:**
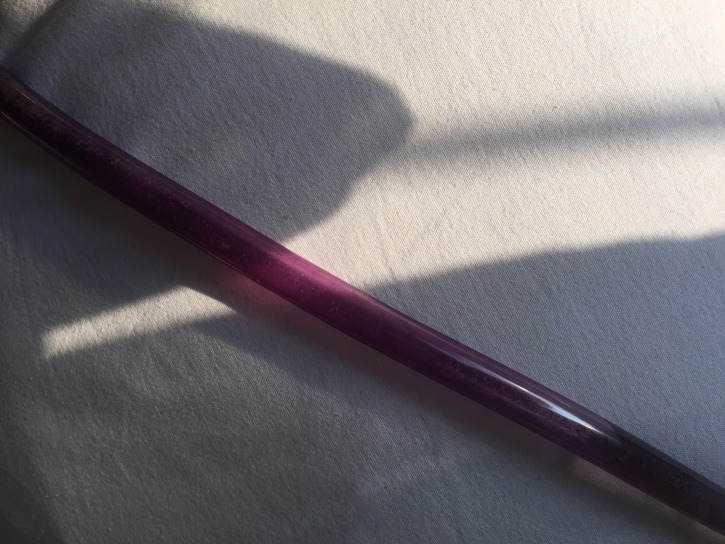
Purple urine in catheter tube.

The patient’s catheter tubing and bag contained bright purple urine with small amounts of pale colored sediment. Figures 1-2 demonstrate purple-colored urine in the urine bag and catheter tubing, respectively. The patient denied any episodes of purple urine in the past. A urine culture grew Proteus vulgaris resistant to Cefazolin and sensitive to other cephalosporins, trimethoprim/sulfamethoxazole (TMP­ /SMX), and piperacillin/tazobactam. He was started empirically on ceftriaxone that was continued after culture results, and the following day clear yellow urine had accumulated in his urine bag. The blood cultures remained negative, and his leukocytosis resolved. After being given four doses of intravenous ceftriaxone and a discernible improvement in symptoms, he was discharged from the hospital on a three-day course of TMP /SMX to complete the prescribed seven-day course of antibiotics.

Informed patient consent was obtained for treatment of this patient.

## Discussion

Since its identification in 1978, PUBS has been largely reported in the literature through case reports. A keen study of these reports enlightens us about the most common risk factors which include constipation, chronic catheterization, alkaline urine, polyvinylchloride catheter tubing, and significant disability, all of which were prevalent in our patient. Other factors include female sex, nursing home placement, and laxative suppository [1-2].

Here, we report the first case of PUBS associated exclusively with the pathogen Proteus vulgaris. The most common pathogens encountered in the cultures of urine samples of patients with PUBS are Escherichia coli, Pseudomonas aeruginosa, Klebsiella pneumoniae, Proteus mirabilis, Morganella, Providencia rettgeri, and Providencia stuartii. Only one other report of PUBS associated with Proteus vulgaris was found in the literature, which reported a hemodialysis patient with a urine culture positive for multiple organisms: Escherichia coli, Enterococcus faecalis, and Proteus vulgaris [3]. The pathogenesis of PUBS involves metabolism of dietary tryptophan in the gastrointestinal tract to indole, which, in turn, is conjugated to indole sulfate in the liver. From there, oxidation of the molecule to indigo and indirubin produces both blue and red hues that concentrate in catheters; together, they give a purple appearance [4-5]. The condition is benign and frequently described as rare, despite one reported incidence noted as high as 16% [2-3]. Resolution is often seen after changing the tubing of the catheter system and with antibiotic therapy, as was the case with our patient.​

## Conclusions

Purple colored urine is not a clinically irrelevant finding in patients with long-term urinary catheters and should not be overlooked. It may indicate the presence of specific bacteria that have the capacity to change a precursor endogenous substrate to a blue colored metabolite in patients with specific risk factors, such as longstanding physical debility. The list of bacteria that have this capacity is very narrow and includes Escherichia coli, Pseudomonas aeruginosa, Klebsiella pneumoniae, Proteus mirabilis, Proteus vulgaris, Morganella, Providencia stuartii, and Providencia rettgeri.

This information can act as a guide to narrow down the causative organisms and choose appropriate antibiotics for eliminating the same. In addition, normalization of the color of urine can help us to assess the response to therapy, but this finding needs to be validated with additional data, for which ample opportunities are and will be encountered in clinical practice​.​
